# Editorial: Post-COVID-19 cardiovascular sequelae

**DOI:** 10.3389/fcvm.2023.1191953

**Published:** 2023-04-26

**Authors:** Dorina-Gabriela Condurache, Mayooran Shanmuganathan, Zahra Raisi-Estabragh, Betty Raman

**Affiliations:** ^1^ William Harvey Research Institute, NIHR Barts Biomedical Research Centre, Queen Mary University of London, London, United Kingdom; ^2^ Barts Heart Centre, St Bartholomew’s Hospital, Barts Health NHS Trust, London, United Kingdom; ^3^ Division of Cardiovascular Medicine, Radcliffe Department of Medicine, University of Oxford, Oxford, United Kingdom; ^4^ Cardiology Department, Wycombe Hospital, Buckinghamshire Healthcare NHS Trust, High Wycombe, United Kingdom; ^5^ Heart Transplant Department, Royal Brompton and Harefield Hospitals, Harefield, United Kingdom

**Keywords:** COVID-19, cardiovascular disease, vaccine, Long-COVID, myocardial injury

**Editorial on the Research Topic** Post-COVID-19 cardiovascular sequelae

## 1. Introduction

Coronavirus disease 2019 (COVID-19), caused by the severe acute respiratory syndrome coronavirus 2 (SARS-CoV-2) commonly presents as a pulmonary illness but may also affect extrapulmonary organs including the heart (1). Cardiovascular complications have been documented at all stages of the disease, from direct acute myocardial injury during severe acute illness (2) to prolonged cardiovascular involvement in the post-acute phase (3), and more indolent symptoms beyond the acute infection commonly referred to as Long COVID (4). The COVID-19 pandemic, particularly in its early stages, also led to major disruptions in healthcare delivery, which indirectly affected all aspects of cardiovascular care from preventive assessments to acute cardiovascular treatments. Although vaccinations against SARS-CoV-2 attenuated disease severity and mortality after COVID-19, vaccine-induced adverse effects involving the heart such as myopericarditis started emerging (5). The aim of this Research Topic in Frontiers Cardiovascular Medicine was to present research examining these direct and indirect cardiovascular consequences of the COVID-19 pandemic.

## 2. Cardiovascular complications of COVID-19

Growing reports highlight the multisystem manifestations of COVID-19. In particular, existing literature highlights cardiovascular complications of COVID-19 in the acute and early post-acute phase of the illness. In a retrospective cohort study, Lu et al. studied the prognostic impact of multi-organ injury in hospitalised patients with acute COVID-19. They analysed the demographics, clinical variables, and the likelihood of inpatient mortality of COVID-19 patients with combined injury Acute Kidney Injury (AKI) and Acute myoCardial Injury (ACI) and compared them with those with AKI only, acute cardiac injury only, and no injury (NI). Of the 5,896 hospitalized COVID-19 patients, 44% had NI, whilst 19%, 9%, and 28% had AKI, ACI, and AKI-ACI, respectively. They observed that COVID-19 patients with both AKI and ACI had markedly worse outcomes compared to those with NI or each organ injury alone (AKI, ACI). These patients had worse clinical and laboratory variables, markedly worse disease courses, and increased in-hospital mortality. The adjusted odds ratios for in hospital-mortality were 17.1, 7.2 and 4.7 for AKI-ACI, ACI, and AKI, respectively, relative to NI.

Some studies have suggested that there may be persistent cardiovascular involvement in the context of COVID-19, even after apparent recovery from the acute illness. These assertions were mainly based on abnormalities in imaging metrics. Shanmuganathan et al. present a prospective study of middle-aged (median age 56 years) unvaccinated patients (*n* = 23) hospitalised with COVID-19. They observed evidence of myocardial oedema, as demonstrated by significantly elevated myocardial T1 and T2 signals on cardiovascular magnetic resonance (CMR) performed during the acute hospitalisation, in some cases compared to non-COVID-19 and asymptomatic controls matched for cardiovascular risk factors (*n* = 19). Follow up CMR scans performed 6 months after discharge from hospital suggested that acute myocardial oedema tends to normalize over time during the convalescent phase. Ventricular function was preserved throughout. Consistent with these observations, Gao et al. reported no elevation of high-sensitivity troponin I (cTnI) and N-terminal pro-B-type natriuretic peptide (NT-proBNP) or echocardiographic structural and functional abnormalities in survivors of COVID-19 compared with healthy control and risk factor-matched control, almost one year (327 days) after recovery from the acute illness. The authors suggested that any myocardial injury and echocardiographic structural and functional abnormalities observed in the acute phase of COVID-19 infection might be reversible.

Many studies have reported elevated risk of incident cardiovascular events in individuals recovering from COVID-19. In particular, the elevated risk of venous thromboembolism (VTE) after COVID-19 has been consistently demonstrated in different studies and settings (6). These observations have raised the question of potential benefit of prophylactic antithrombin therapy after COVID-19, with current uncertainty about the duration and type of treatment. A study performed in Russia by Motloch et al. investigated the benefits of prophylactic anticoagulation or antiplatelet therapy in Covid-19 patients on long-term survival and cardiovascular outcomes (hospitalization due to pulmonary embolism (PE), myocardial infarction (MI) and stroke). In mid-2020, dipyridamole or prophylactic direct anticoagulation (DOAC) were routinely prescribed in the early post-discharge period (30-days post-discharge) in several medical centres in Russia based on Russian Ministry of Healthcare recommendations. This single centre, retrospective study showed that post-discharge thromboprophylaxis with DOAC/Dipyridamole for 30 days reduced the risk of cardiovascular events and all-cause mortality compared to no anticoagulation, emphasizing the ongoing thromboembolic and inflammatory burden in COVID-19 in the early post-discharge period following the acute phase of the disease.

A growing body of research highlights persistent cardiovascular symptoms in patients apparently recovered from COVID-19, raising questions about the longer-term consequences of infection (7). An observational study conducted by Marques et al. analysed the presence of alterations in cardiac autonomic functioning in 155 patients with long-COVID-19 (asymptomatic or mildly to moderately symptomatic) and compared them with a Covid-19 negative control group (*n* = 94). The study concluded that long COVID clinical group showed reduced heart rate variability (HRV). These findings suggest potential alteration of sympathetic tone following COVID-19 which may help explain some of the symptoms of long COVID-19 as also proposed by Sze et al. in a mini-review in this Topic.

## 3. Post-COVID-19 vaccine cardiovascular complications

Whilst the availability of vaccinations heralded a new phase of the pandemic with a reduction in frequency of severe infections and mortality (8), it was also accompanied by concerns around their safety. In particular a number of case reports and registry data (Wu et al. Cui et al. Sciaccaluga et al.) suggest that myocarditis is a side effect of COVID-19 vaccination. In this series, two case reports have linked both viral-vector based and inactivated SARS-CoV-2 vaccines to incidences of fulminant myocarditis (Wu et al. Cui et al.). Another case report linked mRNA-based COVID-19 vaccine to myocarditis and pericarditis in two young male patients (Sciaccaluga et al.). Shiyovich et al. suggest that the CMR imaging findings of myocarditis following the administration of the BNT162b2 mRNA COVID-19 booster vaccine were relatively mild.

Given concerns raised in such early case reports, a number of researchers undertook a more systematic assessments of this research question. Researchers from the CMR-Center of University Hospital Muenster, Germany (Meier et al.) enrolled 41 healthy volunteers for a CMR-based screening study before and after the third booster vaccination to assess the effect of the booster on the myocardium. 30% of the subjects received mRNA- 1273% and 70% received BNT162b2 for booster. The study showed no significant changes in the myocardial tissue characteristics or function following the third booster vaccination; however, they did report one case of subclinical pericarditis in a female patient. The third booster vaccination significantly raised the SARS CoV-2-IgG antibody titre, and curiously, it did so more in females than in males, according to the results. While reassuring, the size of this study limits its generalisability with regards to real-world prevalence of myopericardial injury following mRNA vaccination.

## 4. Impact of pandemic on cardiovascular healthcare

The COVID-19 pandemic has significantly impacted cardiovascular care across key areas of health care delivery including preventive interventions as well as the management of acute and chronic disease. A study performed in Lombardi, Italy by Ferlini et al. explored the impact of the COVID-19 pandemic on the presentation, time of care, and mortality data of patients with diagnoses of ACS, including ST-elevation myocardial infarction (STEMI) during the second SARS-CoV-2 pandemic spread (November 2020-January 2021) within the “macro-hubs” network implemented by the Lombardy region, to keep the regional healthcare system from being overwhelmed, and to guarantee timely optimal care to patients with acute coronary syndromes (ACS). The observational study included 941 ACS patients and a total of 59 patients (6.3%) presented a concomitant confirmed SARS-CoV-2 infection out of which 42.4% of patients had pneumonia. STEMI was the clinical presentation in 56% of SARS-CoV-2 infected patients.

The study revealed that patients with ACS (STEMI) and positive SARS-Cov-2 (based on the positive nasopharyngeal swab, pulmonary TAC diagnostic for interstitial pneumonia, as a single test or in combination) had a higher GRACE (Global Registry of Acute Coronary Events) score of 139 (IQR 105–158) and a considerably greater rate of in-hospital death than those without infection (16.9 vs. 3.6%), whereas post-discharge mortality was not affected (4.2 vs. 4.1%). This excess mortality risk appeared to be attributable to the presence of concomitant pneumonia (Ferlini et al.). Ferlini et al. reported the centralized model used in Lombardy did not show a negative impact on time to treatment of STEMI patients. Furthermore, almost all patients with ACS received coronary angiography (97%) for STEMI, corroborating the beneficial effect of the organizational strategy adopted.

Contrasting this approach in Italy, Tang et al. observed a decline in percutaneous coronary intervention (PCI) activity during COVID19 pandemic outbreak (January 23, 2020 to April 8, 2020) in Hunan province, China. When compared with prepandemic levels there was a 12.7% reduction in PCI procedures in COVID-19 negative patients. The authors also reported a 10.5% drop of STEMI admissions. Overall, in this study, restructuring health care services during the COVID-19 pandemic outbreak did not appear to significantly adversely influence in-hospital mortality and major cardiac events. The authors suggested that multiple factors might contribute to this decline in admissions of patients with STEMI including misdiagnosis because of complex cardiovascular manifestations under the circumstance of COVID-19 as well as reluctance of symptomatic patients to seek acute medical care due to a fear of catching COVID-19.

There were also changes in uptake of cardiovascular assessments in other areas. Li et al. performed a study using an online ECG platform in China to investigate how COVID19 affected the health-seeking behaviour of patients with various arrhythmias with non-COVID-19 diseases, during and after COVID-19 epidemic. Compared with the same period during pre-COVID years, the number of medical visits decreased during the lockdown (a 38% reduction), followed by a rebound post-lockdown (a 17% increase) and a fall to the baseline level in post-SARS-CoV-2 period. The ECG utilization patterns of patients with arrhythmias exhibited a decrease-rebound-fallback pattern following the COVID-19 lockdowns. Lockdowns had less of an impact on medical visits for illnesses with more severe symptoms, demonstrating a persistent need for healthcare.

## 5. Conclusion

**Figure 1:**
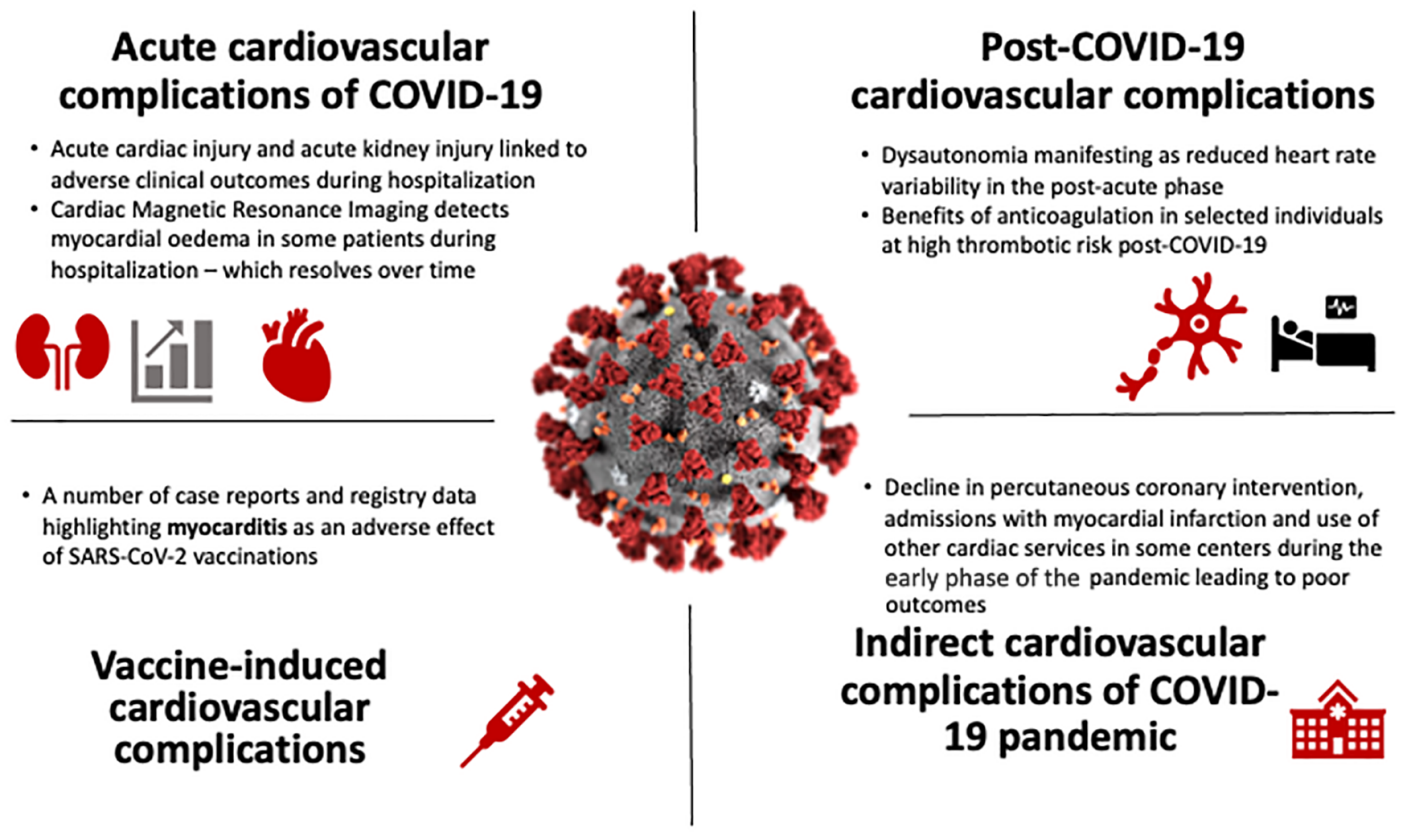
Cardiovascular complications of the COVID-19 pandemic.

In summary, this Research Topic has covered a range of articles from around the world, covering matters relevant to our understanding of the direct and indirect cardiovascular consequences of COVID19 (Figure 1). While acute cardiac manifestations were reported in the early phase of the pandemic, the decreased virulence of evolving SARS-CoV-2 variants and the protective effects of vaccines and acquired immunity from natural infections have reduced the rates of severe complications and mortality from COVID-19. However, one must be vigilant of the potential of even mild COVID-19 to cause ongoing symptoms (e.g., dysautonomia) and of the infrequent presentation of myopericarditis after COVID-19 vaccinations. The high cardiovascular mortality in the early pandemic period could have also resulted from a failure of health care systems to rapidly adapt to health care needs during a global health crisis and reinforces the need for greater investment into agile services in preparation for future pandemics.
